# Distribution of the Number of Paths in Two-Dimensional Directed Percolation

**DOI:** 10.3390/e27101008

**Published:** 2025-09-26

**Authors:** Leon Seeger, Alexander K. Hartmann

**Affiliations:** Institut for Physics, University of Oldenburg, 26111 Oldenburg, Germany

**Keywords:** percolation, lattice, simulations, Monte Carlo algorithms, large deviations, bias, paths

## Abstract

In a percolating system, there are typically exponentially many spanning paths. Here, we study numerically, for a two-dimensional L×L diluted system, restricted to percolating realizations, the number *N* of directed percolating paths. First, we study the average entropy 〈S〉=〈logN〉 as a function of the occupation density *p* and compare with mathematical results from the literature. Furthermore, we investigate the distribution P(S). By using large-deviation approaches, we are able to obtain P(S) down to the very low-probability tail reaching probabilities as small as $10^{-300}$. We consider the percolating phase, the (typically) non-percolating phase, and the critical point. Finally, we also analyze the structure of the realizations for some values of *S* and *p*.

## 1. Introduction

Percolation is a widespread phenomenon [1,2,3,4] where one asks, e.g., whether a porous material allows for the flow of liquids through the bulk of a probe. It finds applications in many fields [3,5] such as graph theory, magnetic systems, analysis of algorithms, social systems, economy, and even games [6]. In general, one distinguishes *active* or *occupied* sites from *non-active* or *unoccupied* sites. Note that for the case of the porous material, the occupied sites are the pores, which are actually not occupied by the material. Often, one considers given realizations on finite-dimensional lattices, like hyper-cubic ones, and asks whether a percolation path exists or not, for example, from one side of the system to an opposite site, or from one specific site to another specific site. When studying ensembles of realizations, parameterized, e.g., by the density of occupied sites, a phase transition between the percolating phase, with high occupation density, and a non-percolating phase, with low occupation density, emerges. In physics, the properties of these phase transitions are of interest, as described, e.g., by critical exponents.

Nevertheless, in the case of a percolating system, there usually exists not only one percolating path but many, and in most cases, exponentially many [7]. Since the number *N* of paths, in particular, its logarithm, i.e., the entropy *S*, plays an important role in statistical mechanics, it makes sense to ask how *S* behaves in the different phases. Note that this means that in both phases, the analysis is limited to the percolating realizations, which are frequent in the percolating phase and rare in the non-percolating phase. Because the percolation model exhibits disorder, which means that there are many random realizations, *S* itself is a random quantity. This means that *S* is described by a distribution P(S) where its functional form might depend, e.g., on the fraction *p* of occupied sites. In particular, we test whether the observed distributions are broadly compatible with Gaussian distributions.

The behavior of the average entropy has received some interest. Recently, for the case of *directed* percolation [8,9] on hypercubic lattices, the set of paths of a certain length *l* starting at the origin was considered [10]. It was shown that for a large enough value of *p*, i.e., deep enough in the percolating phase, the typical number of distinct paths of length *l* for a given realization grows according to

(1) $$N∼e^{\alpha (p)l}$$

in the limit of large path lengths, where

(2) α(p)=log(pz),

and where *z* is the maximum number of possible directions a directed path can proceed. Note that for the so-far cited samples for d+1 dimensional systems, i.e., *d* spatial and one “time” direction, the case z=2d+1 was considered. For the present model in d+1=2 dimensions (see below), we have z=2. Note that in earlier work [11,12], it was only shown that logpz is an upper bound and becomes tight for higher dimensions d≥4, in the limit of large occupation p→1.

In this work, we study, by computer simulations, two-dimensional directed percolation. By using a dynamic-programming algorithm, we determine, for each percolating realization, the number N>0 of percolating paths. By averaging over the disorder, this yields the average entropy 〈S〉=〈logN〉 as a function of the occupation density *p*. But our main effort is devoted to the full distribution P(S). By using Markov chain large-deviation approaches, where the configurations of the Markov chain are the disorder realizations [13,14,15], we are able to obtain P(S) down to exponential small probabilities, such that we can also determine the *large-deviation rate function* ϕ(S), which is a fundamental quantity in large-deviation theory [16,17]. In particular, we study P(S) and ϕ(S) in the percolating and non-percolating phases, as well as at the critical point. Note that even in the non-percolating phase, rare percolating realizations exist. These we can sample numerically also by using the Markov chain approach.

The paper is organized as follows. In the following section, we present the model. Next, we explain the dynamic programming algorithm to calculate *N* and the large-deviation approach to obtain P(S) also in the tails where the probabilities are as small as $10^{-300}$. Then, in Section 4, we show our results for the average 〈S〉 and the distribution P(S) and present some analysis of the structure of the disorder realizations in the different phases. Finally, we summarize our results and outline some possible future directions of research.

## 2. Model

We define, for a two-dimensional lattice of size L×L, a *realization* of the disorder as a set $x=\{x_{i,j}\}$ of occupation values with $x_{i,j}=0,1$ for i,j∈{1,2…,L} with

(3) $$x_{i,j}=\left\{\begin{array}{cc} 1 & \mathrm{if}\;\mathrm{site}\;(i,j)\;\mathrm{is}\;\mathrm{occupied}\\ 0 & \mathrm{else} \end{array}\right.\;.$$

**Figure 1:**
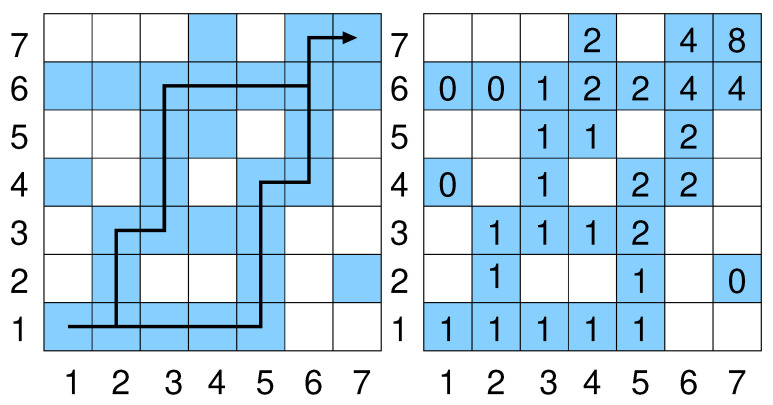
(**left**) An example system with size L=7. The shaded sites are occupied; the white sites are empty. Two percolating paths are shown, i.e., connecting (1,1) with (L,L). (**right**) For each occupied site the number N(i,j) of paths from (1,1) to (i,j) is shown, as given by Equation (8). In total, there are N(L,L)=8 percolating paths.

For the present model, for implementational simplicity, we consider, below, paths of occupied sites between the lower left and the upper right corner. Thus, we choose that these corners are always occupied, i.e., $x_{1,1}=x_{L,L}=1$. Each other site is independently occupied with probability *p* with 0≤p≤1. An example for such a realization is shown in Figure 1.

An occupied site (i,j) is considered *connected* to a neighboring site (i−1,j), (i,j−1), (i+1,j), (i,j+1) if the neighboring site is occupied as well. A *directed path* from (i,j) to $\left(i+l_{x},j+l_{y}\right)$ with $0\leq l_{x}\leq L-i$ and $0\leq l_{x}\leq L-j$ is a sequence $\left(i_{1},j_{1}\right),\left(i_{2},j_{2}\right)$, …, $\left(i_{l},j_{l}\right)$ of length $l=l_{x}+l_{y}$ of connected sites such that the position of the sites increases by one in the *x* or *y* direction in each step, i.e., $\left(i_{n+1},j_{n+1}\right)=\left(i_{n}+1,j_{n}\right)$ or $\left(i_{n+1},j_{n+1}\right)=\left(i_{n},j_{n}+1\right)$. Two examples for such directed paths are shown in Figure 1.

A disorder realization which contains a directed path of length l=2L−1 from (1,1) to (L,L) is called *percolating* (or *spanning*), and also the path is called percolating. Thus, the indicator function, whether a realization is percolating or not, is a random variable. We denote by $P_{\mathrm{perc}}$ the probability that a realization is percolating and estimate it below numerically as a function of occupation probability *p*.

For a percolating realization, there might be several directed percolating paths. Note that different paths might share some sites, but they cannot share all sites. We denote, for each disorder realization,

(4) N=Numberofpercolatingpaths

and the *entropy*

(5) S=logN.

We consider the entropy as the quantity of interest here, because on average, it will not be dominated by rare realizations with extreme large number of paths, in contrast to *N*. Since *S* is a disorder-dependent random quantity, it is described by a distribution $P_{L,p}\left(S\right)$. Here, we explicitly include the dependence on the system size *L* and on the occupation density *p*, which is omitted from Section 3 for simplicity of notation. Note that this distribution is conditioned to those disorder realizations which are percolating.

We denote the related disorder average by 〈…〉, in particular, 〈S〉 is the average entropy constrained to the percolating realizations, i.e., where N>0 and, hence, S≥0.

From Equations (1) and (5), we expect α(p)=〈S〉/l and define correspondingly, for given size *L* and given value of *p*, the finite-size entropy per length as

(6) α(p,L)=〈S(p,L)〉/(2L−1).

Many random quantities exhibit the *large-deviation property* [16,17], i.e., the distribution is exponentially small in the system size, here, the (half) length *L* of the path, with *rate function* $\phi_{p}$. For *S*, this means it has the functional form $P_{L,p}\left(S\right)\propto \exp\left(-L\phi_{p}\left(S\right)+o\left(L\right)\right)$ where o(L) denotes contributions which are sub-linear in *L*. Correspondingly, we define the empirical rate function

(7) $$\phi_{L,p}\left(S\right)=-\frac{1}{L}\log P_{L,p}\left(S\right)\;.$$

Note that in our analysis, we have shifted the rate function such that its minimum occurs at a ϕ-value of 0, i.e., we have subtracted the minimum of $-\frac{1}{L}\max_{S}\log P_{L,p}\left(S\right)$. This is commonly performed for easier comparison, since the actual $\phi_{p}\left(S\right)$ must exhibit a minimum at zero, to allow for normalization and for nonzero probabilities.

## 3. Methods

A realization $x=\{x_{i,j}\}$ of the disorder with size L×L can be generated directly by iterating through all sites (i,j), except (1,1) and (L,L), and independently assigning $x_{i,j}=1$ with probability *p* and $x_{i,j}=0$ with probability 1−p. Thus, the probability for a given realization is $Q\left(x\right)=p^{n_{1}-2}{\left(1-p\right)}^{n_{0}}$, where $n_{1}$ is the number of sites assigned with 1, including the two always occupied sites (1,1) and (L,L), and $n_{0}=L^{2}-n_{1}$ the number of sites assigned with 0.

For a given realization *x*, we define N(i,j) for i,j∈{1,2,…,L) as the number of directed paths from site (1,1) to site (i,j). Since site (1,1) is always occupied in our model, we have N(1,1)=1. The number of directed paths to a given site (i,j) is zero if the site is not occupied ($x_{i,j}=0$). Else it is the sum of the number of paths to sites “incoming” to (i,j), i.e., which can precede (i,j) on directed paths. Thus, we have

(8) $$N\left(i,j\right)=\left\{\begin{array}{cc} x_{i,j}N\left(i-1,0\right) & \mathrm{if}\;j=0\\ x_{i,j}N\left(0,j-1\right) & \mathrm{if}\;i=0\\ x_{i,j}\left(N\left(i-1,j\right)+N\left(i,j-1\right)\right) & \mathrm{else} \end{array}\right.$$

Thus, starting from the lower left, all values of N(i,j) can be calculated, e.g., in a row by row fashion, leading to a running time of $O(L^{2})$. Such an approach is called *dynamic programming* in computer science [18]. An example calculation is shown in the right of Figure 1.

We obtain the number *N* of percolating paths for each realization as N=N(L,L). If N=0, the realization is not percolating, and, therefore, not included in the statistics. For a percolating realization *x*, the obtained entropy is denoted as S(x).

One can obtain an estimate for the distribution P(S) by *direct sampling*. This means that one generates a certain number *K* of realizations, say, $K=10^{6}$, applies the dynamic programming approach Equation (8) to each realization and, finally, calculates S=logN(L,L) for the percolating realizations, respectively. Then, a histogram of the values of *S* can be calculated, which is an approximation of P(S). Clearly, the smallest non-zero frequency one measures in the histogram is at least 1/K, e.g., $10^{-6}$.

To obtain the distribution down to the tails, for probabilities here as small as $10^{-300}$, we employ a large-deviation algorithm [13,14,15] based on Markov chain Monte Carlo sampling. With direct sampling, this would require about $10^{300}$ samples, which is unfeasible. We explain here, for brevity only, the main steps and mention all necessary details relevant for the present simulation.

The basic idea is to generate realizations *x* not according to the original probabilities Q(x), but according to probabilities where the sampling is concentrated in some region of interest, commonly called *importance sampling*. Here, we extend Q(x) by an exponential *bias*, or *tilt*

(9) exp(−S(x)/Θ),

inspired by the Boltzmann distribution controlled by a *temperature-like* parameter Θ. For Θ=∞, the bias is one, i.e., the original distribution recovered. For Θ>0, being small, realizations with small values of *S* are preferred, while for Θ<0, realizations with a large value of *S* appear with higher probability.

We implemented this bias by performing a Markov chain x(t)=x(0),x(1),… of realizations, starting with some initial realization x(0). The initial realization can be a random one, a fully occupied one, etc. We use the Metropolis–Hastings algorithm [19,20] as follows: In each step of the Markov chain, given the current realization x(t), we generate a *trial realization* $x^{\prime }$ by, first, copying x(t) to $x^{\prime }$. Then, we randomly select, $n_{c}$ times, a site (i,j), and each time, we reassign its occupation $x_{i,j}^{\prime }$ according to the original rules, i.e., $x_{i,j}^{\prime }=1$ with probability *p* and $x_{i,j}^{\prime }=0$ else. Thus, the parameter $n_{c}$ controls the number of reassignments performed on x(t) to yield $x^{\prime }$ (For simplicity, we do not exclude that a site is reassigned more than once within one trial generation. This does not violate detailed balance).

Next, we compute the number $N^{\prime }$ of percolating paths for $x^{\prime }$ by using the dynamic programming algorithm Equation (8). If $N^{\prime }=0$, the trial realization is immediately *rejected*, i.e., x(t+1)=x(t). This guarantees that the Markov chain contains only percolating realizations. If $N^{\prime }>0$, the entropy $S^{\prime }=S\left(x^{\prime }\right)=\log N^{\prime }$ is obtained. Let S(t)=S(x(t)) be the entropy of the current realization. Now, the trial configuration is *accepted*, i.e., $x\left(t+1\right)=x^{\prime }$ with probability

(10) $$p_{\mathrm{MH}}=\min\left\{1,e^{-(S^{\prime }-S\left(t\right))/\Theta }\right\}\;.$$

Otherwise, i.e., with probability $1-p_{\mathrm{MH}}$, the trial realization is rejected. This algorithm guarantees, after suitable equilibration, that the sampling of configurations is performed with the total weight $Q_{\Theta }\left(x\right)=Q\left(x\right)\exp\left(-S\left(x\right)/\Theta \right)/Z\left(\Theta \right)$, where $Z\left(\Theta \right)=\sum_{\tilde{x}}Q\left(\tilde{x}\right)\exp\left(-S\left(\tilde{x}\right)/\Theta \right)$ is the normalization.

As a rule of thumb, we attempted to obtain an empirical acceptance rate of the trial realizations of about 50%. We tuned the parameter $n_{c}$ for each temperature-like parameter accordingly. Therefore, for small |Θ|, one has smaller values of $n_{c}$, while in the limit |Θ|→∞, where $p_{\mathrm{MH}}\to 1$, one can reassign all entries of x(t), which corresponds to direct sampling restricted to percolating realizations.

**Figure 2:**
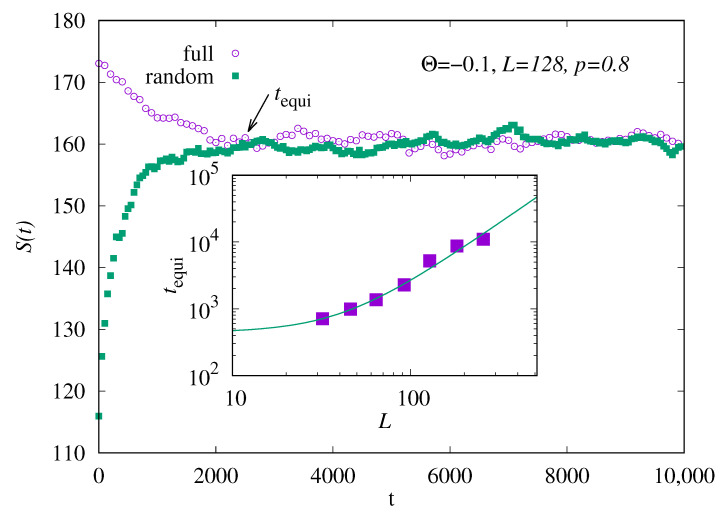
Main plot: Entropy *S* as a function of the Monte Carlo time *t* for one run with system size L=128, p=0.8 and temperature-like parameter Θ=−0.1, for two different initial realizations. Inset: equilibration time $t_{\mathrm{equi}}$ (see text) as a function of system size *L* for Θ=−0.1. The line shows a fit to the function shown in Equation (11).

Equilibration, i.e., approximate convergence of the Markov chain to the desired measure, can be easily monitored by starting the Markov chain with different initial realizations, for example, one run with a random but percolating realization, and another run with a completely occupied realization. Then, one monitors S(t) for both runs. We consider the Markov chain to be equilibrated, i.e., long enough to sample *S* according to the desired Boltzmann distribution, when the different time series agree within fluctuations. The corresponding time we denote by $t_{\mathrm{equi}}$. An example is shown in Figure 2 for L=128 and Θ=−0.1. Here, equilibration is achieved for about $t_{\mathrm{equi}}=2300$. To obtain an impression how the equilibration time depends on the system sizes, we have averaged the equilibration time for 10 independent runs and repeated the simulation for six other system sizes L∈[32,256]. The resulting slightly averaged $t_{\mathrm{equi}}\left(L\right)$ is shown in the inset of Figure 2. We have fitted a function

(11) $${\tilde{t}}_{\mathrm{equi}}\left(L\right)=t_{0}+cL^{h}$$

to the data, resulting in an exponent h=1.9(2), which means that the equilibration time roughly grows quadratically with the system size. For other values of Θ, where |Θ| is larger, the results look similar, as they do for other values of *p*, but equilibration is achieved faster and *h* is somehow smaller.

Thus, for long enough simulations, one can sample realizations *x* and corresponding values of *S*. They can be collected in histograms, which approximate the biased distributions $P_{\Theta }\left(S\right)$.

One can easily show [13] that the desired unbiased distribution can be obtained by $P\left(S\right)=P_{\Theta }\left(S\right)\exp\left(S/\Theta \right)Z\left(\Theta \right)$. This holds for any value of Θ, but the temperature value determines in which interval data are actually available. Thus, one has to perform simulations typically for several dozens of values of Θ or more, to cover a large range of values of *S*. Note that the values Z(Θ) can be determined, e.g., by exploiting $P_{\Theta_{1}}(S)\exp\left(S/\Theta_{1}\right)Z\left(\Theta_{1}\right)$=P(S)= $P_{\Theta_{2}}(S)\exp\left(S/\Theta_{2}\right)Z\left(\Theta_{2}\right)$ for pairs $\Theta_{1},\Theta_{2}$ such that the generated histograms for *S* overlap [13]. This fixes all relative normalization factors, and the final normalization can be obtained by using normalization of P(S). Alternatively, one can apply the multi-histogram rescaling approach [21], where, also, a convenient Python (Vers. 3 and upwards) tool [22] exists. This allows one to obtain P(S) over many decades in probability.

This approach is rather general and has been used, e.g., to study non-equilibrium processes like measuring the work distribution for the Ising model [15] or unfolding RNA secondary structures [23], the Kardar–Parisi–Zhang model [24], and traffic flows [25]. Also, like in the present work, large-deviation properties of equilibrium problems have been studied, e.g., properties of random graphs [26,27] of biological sequence alignment [13,28], or to obtain the partition function of the Potts model [29].

## 4. Results

**Figure 3:**
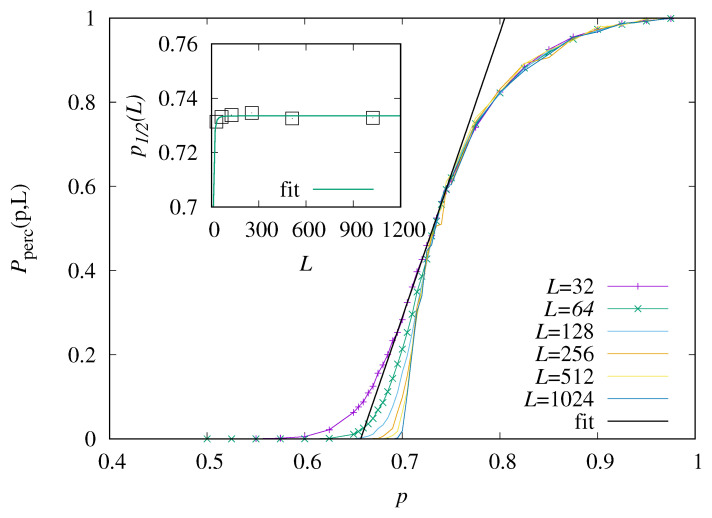
Estimated percolation probability as function of *p*, for varying system sizes *L*. The line shows the fit to Equation (12) for L=32. The inset shows the estimated critical points $p_{1/2}$ as function of system size *L*.

To find out where regions of interest are, first, we have performed direct-sampling simulations, to establish the percolation phase diagram. This means that we generated $10^{4}$ realizations of the disorder and measured, each time, the number *N* of paths. If N>0, the realization is percolating. From these data, we have estimated the percolation probability $P_{\mathrm{perc}}$ as a function of the occupation probability *p*, for varying system sizes L=32,…,1024. The result is shown in Figure 3.

One observes that for small values of *p*, almost all relevant realizations do not percolate, while for large values of *p*, most do. For increasing size *L*, the functions become steeper near p=0.7. Note that even close to p=1, there is still a finite probability that a realization does not percolate, because the directed paths run from the lower left to the upper right corner, i.e., few non-occupied sites close to these corners are sufficient to prevent a percolating path.

We determined the percolation threshold as the value of *p* where $P_{\mathrm{perc}}=1/2$ in the limit of infinite system sizes L→∞. The choice of the value 1/2 is somehow arbitrary; we expect the results not to change much, except for choices too close to 1, which would define almost all realizations as non-percolating. For actually obtaining the critical value, we fit the linear function

(12) $$\tilde{P}\left(p,L\right)=0.5+m\times \left(p-p_{1/2}\left(L\right)\right)$$

with parameters *m* and $p_{1/2}\left(L\right)$ in the range of *p* values where P(p,L) is close to 0.5. The value of $p_{1/2}\left(L\right)$ is taken as finite-size estimate of the percolation point. Finally, we extrapolate to infinite system size by fitting a function ${\tilde{p}}_{1/2}\left(L\right)=p_{c}+cL^{-f}$ to the data, as shown in the inset of Figure 3. This results in the estimate $p_{c}=0.7335(8)$ of the critical point. Since the variation of $p_{1/2}\left(L\right)$ as function of *L* is very small, the obtained fit value for *f* about 3 has an error bar of the same size and, therefore, carries not much information. The value found here is near the result $p_{c}=0.7054850(15)$ obtained by series expansion [30] for a slightly different model. In [30], not only one one target site (L,L) was considered, but instead all target sites (x,y) with x>0, y>0 and x+y=2L. Note that this difference leads also to a different percolation criterion.

Next, we analyze, for those realizations where N>0, the entropy α(p,L) per length from Equation (6). To determine the limiting value of α(p) for infinite system, we performed fits to power laws

(13) $$\tilde{\alpha }\left(p,L\right)=\alpha \left(p\right)+aL^{-b}\;.$$

**Figure 4:**
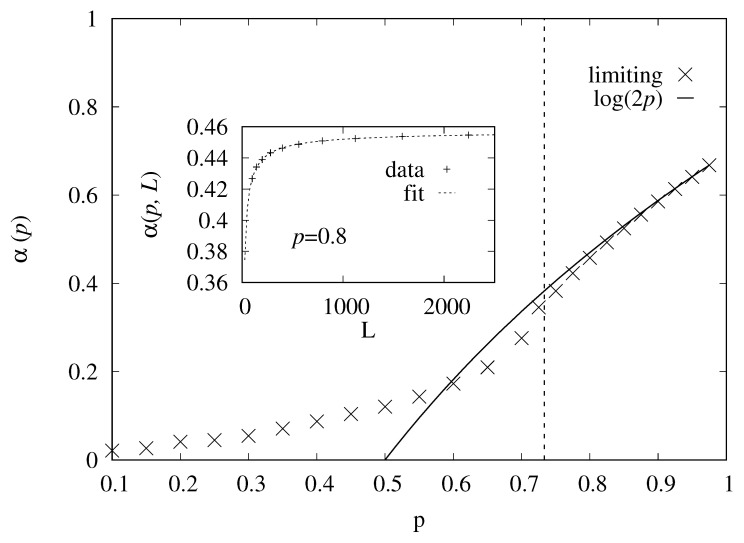
The extrapolated value of α as a function of the site-occupation probability *p*. The solid line shows the analytical result from Equation (2). The vertical dotted line indicates the critical point $p_{c}$. The inset shows an example for the extrapolation as function of the system size *L*, for the case p=0.8.

An example for such an extrapolation is shown in the inset of Figure 4. The main plot shows the extrapolated value α(p) as a function of the occupation probability *p*. One observes that, indeed, for p→1 the asymptotics of Equation (2) is approached, while for smaller values of *p*, it is indeed an upper bound within the percolating phase $p>p_{c}$.

We can use the Markov chain simulation without bias, corresponding to Θ=∞, to generate just percolating realizations even deep in the non-percolation phase $p<p_{c}$. For this purpose, we started each Markov chain with a fully occupied realization, and exploited the fact that only moves are accepted that generate still percolating realizations. Thus, after equilibration, we can again measure *N* and the average entropy. This is restricted to smaller system sizes L≤256, due to the higher numerical effort. Note that for p≤0.5, the size dependence of *L* was rather weak but noisy, so we have used a fit to Equation (13) with fixing a=b=0. One can see in Figure 4 that α(p) becomes a bit steeper near the percolation transition but then becomes almost flat for p→0. Still, the entropy per length (remember that it is an average constrained to the percolating realizations) remains finite for all values of *p*. Naturally, the function log(2p), which estimates α(p) only in the upper percolating phase, will vanish at p=0.5 and cannot be an upper bound for smaller values of *p*.

Next, we consider the distribution P(S) of the entropy over the realizations. Some realizations may exhibit many paths, while others few. Also, the functional form of the function may depend on the phase, whether it is percolating or not. Here, we check, in particular, whether the distribution appears to be (almost) Gaussian or not. Here, to reach the tails of the distributions, we have used the data from the large-deviation Markov chain simulations, with the bias Equation (9). Since these Markov chain simulations require a much larger effort than direct sampling, we have restricted the system sizes to L≤256.

**Figure 5:**
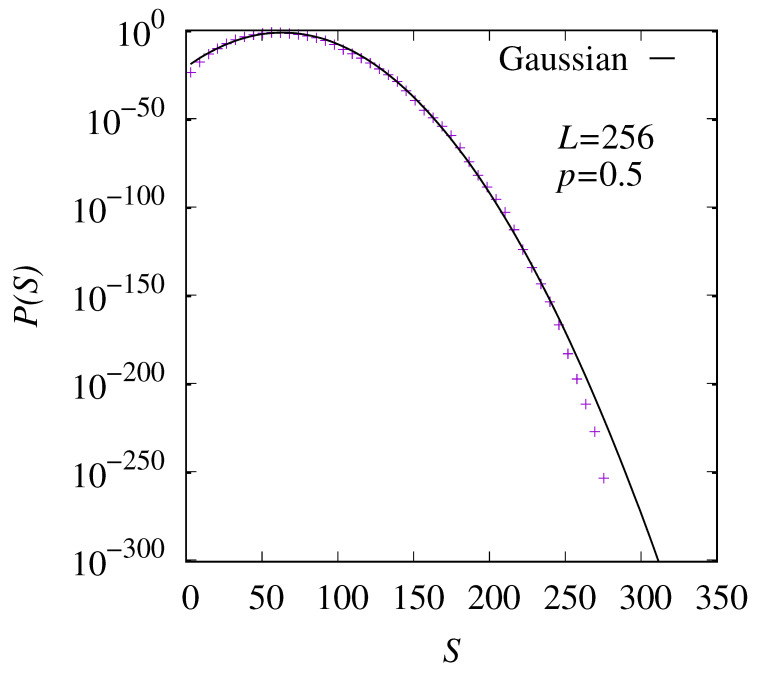
The entropy distribution P(S) for the case p=0.5 and size L=256. The line represents a fit to a Gaussian.

In Figure 5, P(S) is shown in the non-percolating phase at p=0.5 for system size L=256. Note that the choice of $p=0.5<p_{c}$ is rather arbitrary; it is just one representative point in the non-percolating phase. By using the large-deviation approach, we were able to obtain the distribution down to probabilities as small as $10^{-250}$. The distribution is centered about a rather small entropy S≈58, as compared to the maximum entropy $S_{\max}\approx 350$ for this system size. The functional form of the distribution is almost Gaussian, indicated by the result of a fit to a Gaussian which we have performed, finding a peak at $S_{0}=63.4(7)$ and variance $\sigma_{S}^{2}=44.6(4)$.

**Figure 6:**
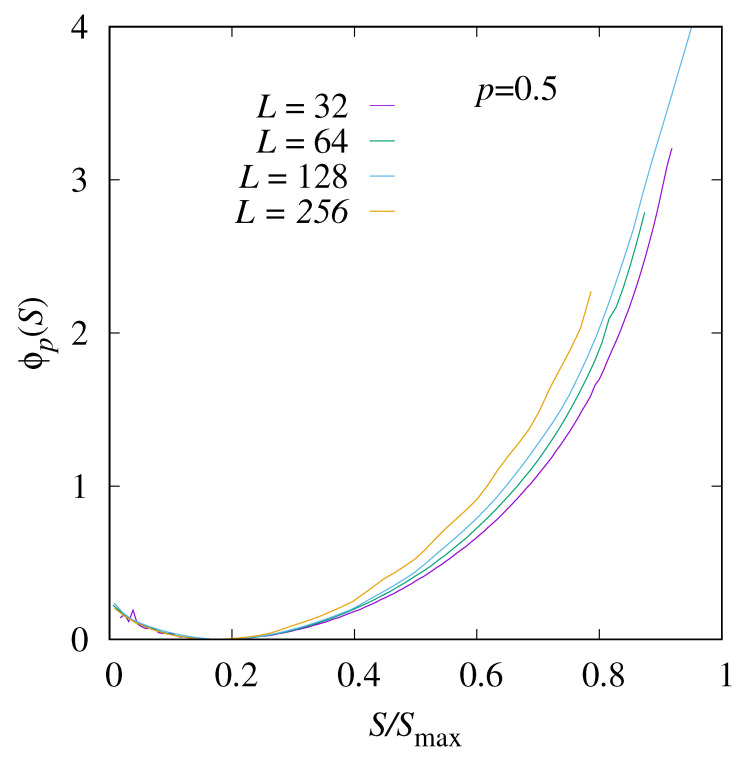
The shifted rate function $\phi_{p}\left(S\right)$ for $p=0.5<p_{c}$ and four system sizes, L=32,64,128, and 256.

Next, we show the empirical shifted rate function Equation (7) in Figure 6 for different system sizes *L*. In the left part, for small values of *S*, the empirical rate functions are basically on top of each other, which means that the limiting rate function exists and is about the same. In total, the Gaussian form is visible for all system sizes. In the right part, one observes considerable finite-size corrections, such that one cannot tell whether a limiting rate function exists and where it runs.

**Figure 7:**
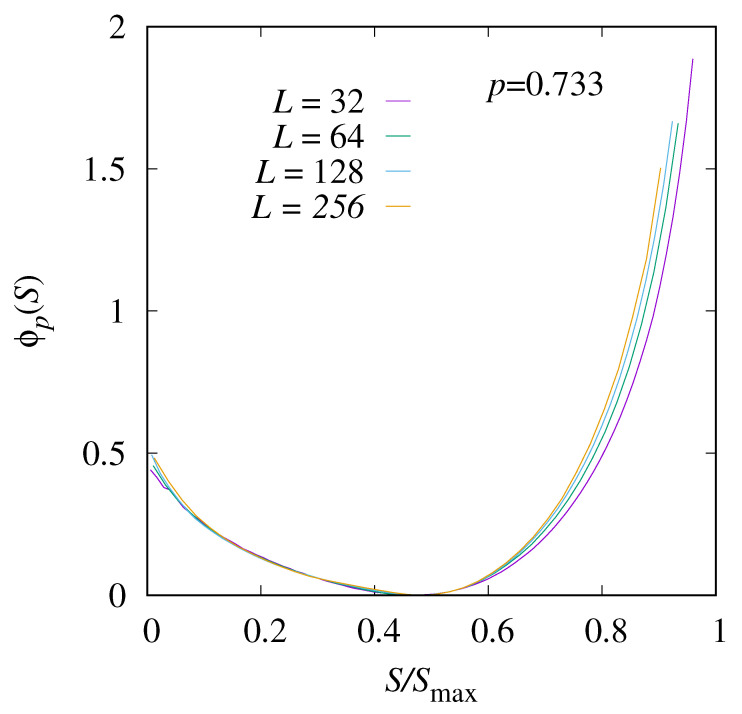
The shifted rate function $\phi_{p}\left(S\right)$ for $p=0.733\approx p_{c}$ and four system sizes L=32,64,128 and 256.

The corresponding rate-function results near the critical point, at p=0.733, are shown in Figure 7. The position of the minimum has shifted to a larger value of $S/S_{\max}$ near 0.5, which is natural, because a higher occupation allows for more paths. The overall functional form is not Gaussian anymore. In the interval between 0 and the minimum, only few changes with the system size are visible, and the data should represent the actual limiting rate function. For larger values of *S*, again, some finite-size corrections are visible and one cannot observe a convergence so far.

**Figure 8:**
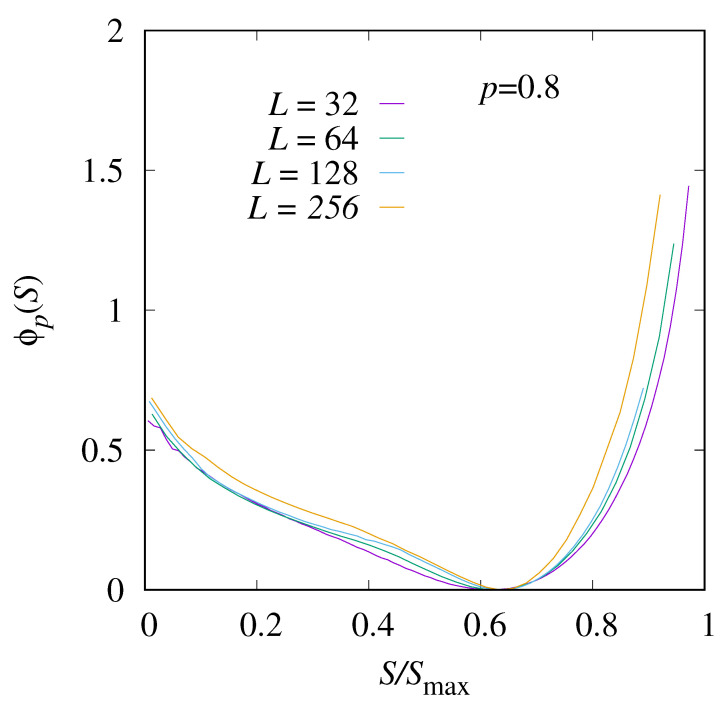
The shifted rate function $\phi_{p}\left(S\right)$ for $p=0.8>p_{c}$ and four system sizes, L=32,64,128, and 256.

The rate function for a value of *p* in the percolating region, here, p=0.8, is shown in Figure 8. Here, the typical value of $S/S_{\max}$, i.e., the minimum position of the rate function, appears for an even higher value of *S*. The functional form of the distribution is very different, in particular, for $S/S_{\max}\in \left[0.2,0.6\right]$, the function seems to behave almost linearly, corresponding to an exponential distribution. Nevertheless, the finite-size corrections are even stronger, so a definite statement about the functional form of the limiting rate function $\phi_{p}\left(S\right)$ cannot be made.

**Figure 9:**
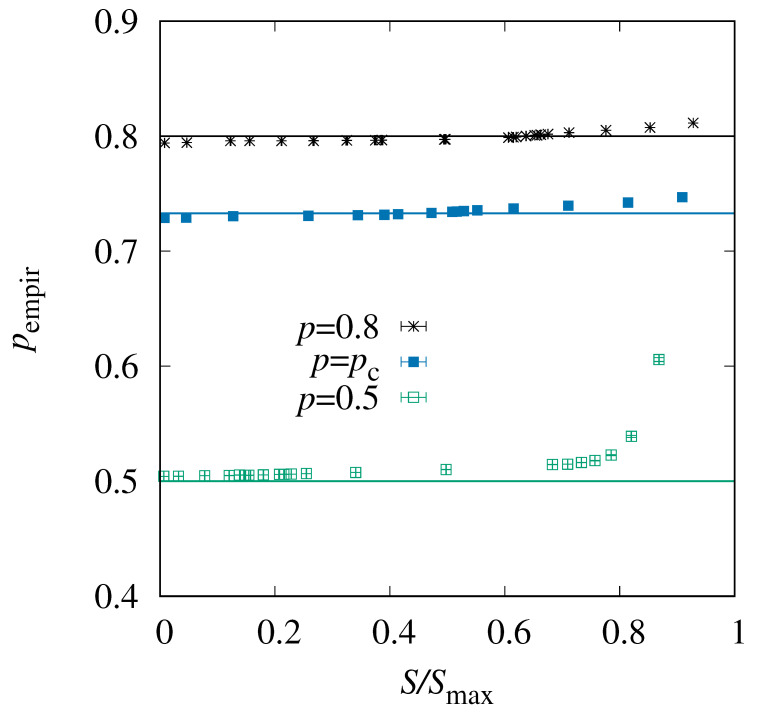
The empirical density $p_{\mathrm{empir}}$ of occupied sites as a function of the normalized entropy $S/S_{\max}$, for system size L=128. The horizontal lines indicate the values of *p*, respectively.

Finally, we consider the actual realizations $x=\{x_{i,j}\}$ and try to establish how they influence their values of *S*. In particular, for any value of *p*, each realization exhibits an individual empirical density $p_{\mathrm{empir}}=\sum_{i,j}x_{i,j}/N$ of actually occupied sites. A natural question to ask is whether $p_{\mathrm{empir}}$ influences the observed entropy *S*, i.e., whether there is a correlation. One would expect, following the *Fortuin–Kasteleyn–Ginibre* inequality [31], that realizations with more occupied sites exhibit more paths, i.e., a higher value of *S*. To verify this, we have, for each temperature Θ, individually recorded and averaged *S* as well as $p_{\mathrm{empir}}$ over all obtained realizations. The result is shown in Figure 9. One observes that for having larger entropy, more sites are actually occupied. For $p=0.8>p_{c}$ and $p=p_{c}$, small values of *S* correspond to values $p_{\mathrm{empir}}<p$, while it is the other way around for large values of *S*. The change of $p_{\mathrm{empir}}$ with *S* is rather moderate. On the other hand, for the same value of *S*, the empirical occupation increases considerably with *p*. For the case $p=0.5<p_{c}$, all empirical, actually observed occupation densities are larger than *p*, which makes sense, because one needs a certain number of occupied sites to have a spanning path at all. Interestingly, for most values of *S*, the value of $p_{\mathrm{empir}}$ is only slightly larger than 0.5 and well below $p_{c}$. This means the occupation is much lower than necessary, on average, for spanning paths, Thus, the occupied sites have to be concentrated in some region, to allow for a percolating path even with an overall small occupation density. We have tested this explicitly.

**Figure 10:**
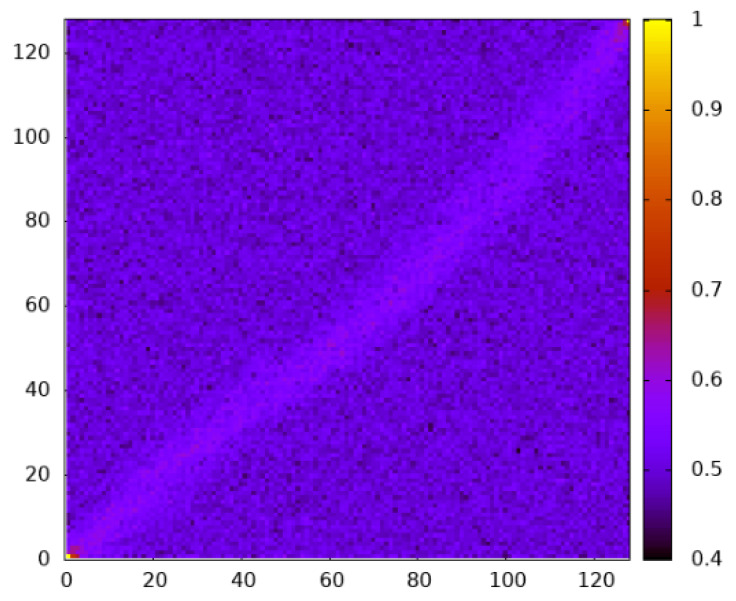
Heat map showing the empirical occupation per site for percolating realizations taken at $\Theta =10^{4}$ for L=128 and p=0.5.

In Figure 10, the average site-dependent empirical occupation values are shown for 9800 independent realizations taken from the MCMC simulation at almost infinite temperature $\Theta =10^{4}$. As usual, these realizations are conditioned to be percolating ones. Still, the high temperature means that the average entropy $S/S_{\max}=0.186$ is typical within the set of conditioned realizations. One observes that the occupation near the diagonal is slightly higher. This explains the existence of percolating paths with a considerable entropy, although the overall empirical occupation is smaller than the percolation threshold.

**Figure 11:**
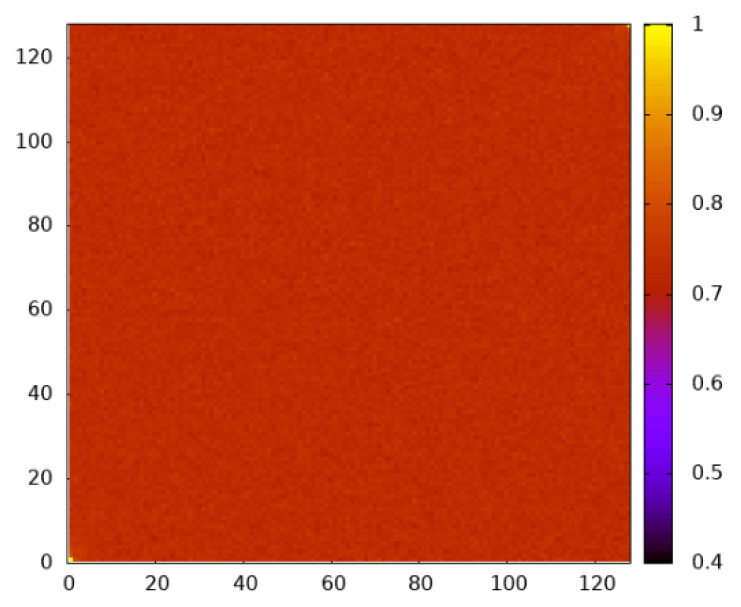
Heat map showing the empirical occupation per site for percolating realizations taken at $\Theta =10^{4}$ for L=128 and $p=p_{c}$.

On the other hand, for the case $p=p_{c}$, apart from the fact that the site-dependent occupation is near 0.733, the typical configurations do not exhibit any spatial structure, as shown in Figure 11. We observed this also for other values of *S*, as obtained from the biased sampling. For the case within the percolation regime, p=0.8, also, no spatial structure could be observed.

## 5. Summary, Discussion, and Outlook

We have studied by computer simulations the number of percolating paths, in particular, the corresponding entropy *S*, for two-dimensional directed percolation. The number grows exponentially with the path length l=2L−1 in the limit of large *l*, which can be written as $e^{\alpha (p)l}$. Our results confirm this scaling according to Equation (2) in the limit p→1.

By using a large-deviation approach, we have been able to obtain not only the disorder-averaged entropy 〈S〉 but also the distribution P(S). The distributions exhibit an almost Gaussian distribution in the non-percolating region $p<p_{c}$, while they have a very non-Gaussian form above the percolation threshold. From the study of the rate function, one observes one part which appears to be linear, i.e., an exponential distribution. Interestingly, this is quite different to that found [24] for the related model of directed polymers in random media (DPRM). The reason is probably that for the directed polymers, a Gaussian distribution of the site weights and a finite temperature were considered, so, also, higher energy sites may be contained in the paths. Here, in contrast, the occupied and non-occupied sites correspond to DPRM with a bimodal (Bernoulli) randomness in the T→0 limit.

In the percolating regime $p>p_{c}$, realizations with large value of *S* have a slightly higher empirical occupation than *p*, while it is the other way around for small values of *S*. Still, the occupied and non-occupied sites appear to be uniformly distributed within a realization. Below the percolating regime, basically all realizations that exhibit percolating paths, i.e., a nonzero value of *S*, exhibit a higher empirical occupation than *p* but smaller than $p_{c}$. This is possible because the occupied sites appear with higher probability near the diagonal, i.e., are concentrated within the realization.

For future studies, in would be of interest to also consider higher dimensions *d*. Here, the Fukushima–Junk bound [10] is expected to be tight, which could be visible for a larger range of values of *p* than in the present work. Still, nothing is known about the distribution P(S). Other percolation models, particularly non-directed ones, would also be of interest. But here, the number of percolating paths is not so straightforward to define, nor to obtain. In this case, one could consider the number of shortest percolating paths instead. Finally, it would be desirable if our work motivates analytical studies with respect to the distribution P(S), in particular, in the large-deviation regime.

## Data Availability

The original contributions presented in this study are included in the article. Further inquiries can be directed to the corresponding authors.
